# Subsurface drip irrigation reduces weed infestation and irrigation water use while increasing inflorescence and cannabinoid yield in an outdoor tunnel *Cannabis sativa* L. production system

**DOI:** 10.1186/s42238-025-00302-x

**Published:** 2025-07-10

**Authors:** Christian Büser, Jens Hartung, Simone Graeff-Hönninger

**Affiliations:** 1 Institute of Crop Science Dept. of Agronomy (340a), Stuttgart, Germany; 2 Institute of Crop Science Dept. of Biostatistics, Stuttgart, Germany

**Keywords:** Water management, Cannabis agronomy, Weed suppression, Irrigation efficiency, Yield optimization

## Abstract

Cannabis (*Cannabis sativa* L.) has served as a valuable medicinal plant for thousands of years and is experiencing a resurgence in cultivation and research due to recent legal changes. However, the resource-intensive nature of cannabis cultivation, particularly water and energy demands, poses significant environmental challenges. Outdoor cultivation in a semi-controlled environment can reduce those energy demands but necessitates irrigation. Drip irrigation (DI) is the most commonly used irrigation method but is often criticized for its susceptibility to water losses through evaporation and the risk of surface runoff. Subsurface Drip Irrigation (SDI) provides a sustainable solution by minimizing evaporation losses while maintaining or increasing yields, thereby enhancing water use efficiency. In this study, we compared the effects of DI and SDI on weed infestation, total water usage, inflorescence yield, and water use efficiency of three CBD-rich cannabis chemotype III genotypes (Kanada, Terra Italia, FED) in an outdoor foil tunnel cultivation system. SDI resulted in a reduction of irrigation water usage by 18.6% compared to DI. Remarkably, weed dry biomass was reduced by 93.2% in SDI. Concomitantly, inflorescence yield increased by 5% and CBD concentration by 9%. Overall, the water use efficiency of inflorescence yield and CBD concentration was significantly higher in SDI than in DI. Our results indicated that implementing SDI instead of DI can significantly decrease irrigation water use and reduce weed infestation while increasing inflorescence and CBD yield, thus reducing the environmental challenges associated with cannabis cultivation.

## Introduction

Cannabis (*Cannabis sativa* L.) has a long history of cultivation, with evidence of its use dating back over 5,000 years (Pisanti and Bifulco 2019). In ancient civilizations, cannabis was highly valued for its versatility, serving medicinal and practical purposes (Pisanti and Bifulco 2019). The medicinal value of cannabis is due to the plant’s diverse phytochemical properties, which are classified into five primary chemotypes distinguished by their cannabinoid profiles (Salamone et al. 2022).

Each chemotype supports distinct uses, contributing to cannabis’ agricultural and cultural significance (Jin et al. 2021; Salamone et al. 2022). Recent legal changes in cannabis cultivation have created unprecedented demand, prompting its growth across various agricultural, technological, and scientific fields. However, scaling production to meet this demand introduces environmental challenges (Zheng et al. 2021). Cannabis cultivation, especially in indoor systems, is resource-intensive, leading to significant greenhouse gas emissions from temperature and humidity control and reliance on artificial lighting, as well as substantial water usage that can exceed two liters per plant and day, depending on cultivation system and genotype (Dillis et al. 2020; Summers et al. 2021; Zheng et al. 2021; Duong et al. 2023; Desaulniers Brousseau et al. 2024).

Furthermore, the environmental impact of fertilizer use in cannabis cultivation has been more frequently discussed in recent years (Chien et al. 2009). Some techniques aimed at increasing yields and cannabinoid concentrations in cannabis cultivation are supported primarily by anecdotal evidence. For example, while increased fertilizer usage has been shown to enhance yield, it has not consistently demonstrated improvements in cannabinoid concentration (Bevan et al. 2021; Song et al. 2023; Hershkowitz 2024). Furthermore, recent research has found no significant effect of nutrient flushing, a common practice in the cannabis industry aimed at increasing yield and cannabinoid concentration, on yield. Changes in metabolite levels were small and varied depending on the cultivar and plant organ tested (Saloner et al. 2024). This fact and the understanding that mineral fertilizer resources are not unlimited and shortages may occur in the future (Alewell et al. 2020) have prompted efforts to increase fertilizer use efficiency. In recent years, one approach has been to utilize advances in nanotechnology (Singh 2016; Babu et al. 2022). Nanofertilizers are defined as fertilizers whose particle size is between 1 and 100 nm in at least one dimension (Singh 2016). Nanoparticles exhibit unique physiochemical properties due to an increased ratio of surface atoms to core atoms. As surface atoms have more unpaired electrons, they exhibit increased reactivity and are expected to enhance nutrient use efficiency, as less fertilizer may be required to achieve the same results compared to conventional bulk materials (Singh 2016; Kah et al. 2018; Babu et al. 2022). Numerous studies on multiple plant species have investigated the impact of nanostructured fertilizers on plant growth and nutrient efficiency. Yet, the effects of these nanofertilizers vary, showing adverse, neutral, or beneficial outcomes based on their particle composition, size, and concentration (Kah et al. 2018; Reddy et al. 2024; Tripathi et al. 2024). Moreover, only a limited number of studies took place under field conditions, limiting the meaningfulness of these effects in plant cultivation, and the long-term environmental impact of nanoparticles remains yet unclear, questioning the ecological benefit compared to conventional fertilizers (Somasundaran et al. 2010; Kah et al. 2018; Khan et al. 2019).

A practical method to lower the resource use intensity in indoor cannabis cultivation involves transitioning to outdoor cultivation systems. Although these systems can reduce resource intensity, they also present new challenges in sustaining sufficient yield and product quality, especially in pest and disease management. The presence of pathogens like *Botrytis cinerea*, a mold that thrives in humid conditions, complicates outdoor cannabis farming. This pathogen can rapidly spread through crops, reducing yield and quality while highlighting the need for effective pest management strategies (Punja and Ni 2021; Zheng et al. 2021; Buirs and Punja 2024). Foil tunnels represent a significant advancement in outdoor cannabis cultivation, providing a semi-controlled environment with moderate temperature fluctuations and a lower risk of mold infestations, as the inflorescences are not exposed to high rainfall and cold, wet conditions, especially during the fall period. By altering the microclimate, foil tunnels extend the growing season and offer flexibility in planting and harvesting. They also protect plants from direct precipitation, which helps prevent issues like root rot and mold (Lamont 2009; Charles et al. 2024). However, the lack of rainfall necessitates effective and efficient irrigation strategies to meet cannabis water requirements and enhance the overall water use efficiency in the system.

One of the most widely used irrigation methods for watering crops is surface drip irrigation (DI). This technique typically involves plastic tubes perforated at regular intervals and equipped with pressure-compensated drippers, which apply small amounts of water directly to the soil surface at the base of each plant. This approach increases water use efficiency and offers a viable irrigation solution in regions where the cost of water or ecological concerns render traditional surface irrigation methods impractical (Garb and Friedlander 2014). However, even with surface DI’s relatively efficient water delivery, applying water to the topsoil inevitably leads to evaporation losses. This issue is particularly pronounced under high temperatures or when the microclimate within a cultivation system is altered to meet the crops’ demands (van der Kooij et al. 2013; Martínez and Reca 2014).

Approximately 90% of the total irrigated area being irrigated using surface wetting methods, resulting in substantial evaporation losses (Guo et al. 2023). A possible alleviation of this issue is the use of subsurface drip irrigation (SDI). SDI is a technique that delivers water to the crop’s roots at depths of 0.1 to 0.5 m below the soil surface through irrigation tubes, increasing the irrigation water use efficiency (IWUE) by minimizing surface evaporation (Camp et al. 2000; van der Kooij et al. 2013). Many different technical approaches to irrigating below the soil level were tested, such as vertically installed perforated pipes with conventional drippers filling the tube (Martínez and Reca 2014), burying conventional drip irrigation tubes (Sorensen and Lamb 2015; Ardenti et al. 2022), or low-pressure ceramic emitters (Cai et al. 2021). Initially, SDI was mainly used in permanent plantings as fruit orchards or vines, as the tubing might interfere with management practices such as plowing, but advances in no-till agriculture and the technologies used led to further implementation in annual crops (Gao et al. 2014; Sorensen and Lamb 2015; Valentín et al. 2020). In a manifold of studies, it has been shown for multiple crops, including annual field crops, that SDI can significantly reduce water usage without compromising yield levels or crop quality parameters (Camp et al. 2000; Lamm 2002; Martínez and Reca 2014; Bozkurt Çolak 2021, 2023; Yang et al. 2024). An additional advantage of this irrigation technique is that it significantly reduces weed germination and growth in dry topsoil. This offers an eco-friendly alternative to traditional weed control methods, such as herbicides, mulch foil, and mechanical approaches, and facilitates the implementation of conservation agriculture (Lamm 2002; Sorensen and Lamb 2015). Furthermore, through the reduction in topsoil moisture, Ardenti et al. (2022) showed that N_2_O emissions can be reduced by up to 46% in a maize cultivation system when SDI was used compared to conventional surface wetting methods. This irrigation technique, therefore, can increase the resource use efficiency of a cannabis cultivation system by directly reducing resource input and reducing competition for resources while decreasing the required labor (Lamm 2002; Kakraliya et al. 2024).

While SDI is relatively well-studied for multiple crops, to our knowledge, no research has been conducted on its suitability for cannabis within a foil tunnel production system, and the effect SDI has on cannabis cultivation remains elusive. Recent research highlights the strong influence of soil-related conditions and nutrient management on cannabis growth and development. Nutrient availability plays a key role in driving vegetative biomass accumulation and significantly impacts reproductive development, including flower yield and morphogenesis (Bevan et al. 2021; Massuela et al. 2023; Morad and Bernstein 2023; Saloner and Bernstein 2023). Moreover, nutrient supply, especially nitrogen availability, and type of source, as well as soil properties, have been shown to modulate secondary metabolism and the ionome, affecting the biosynthesis of cannabinoids and terpenoids (Bernstein et al. 2019; Shiponi and Bernstein 2021a; Saloner and Bernstein 2022; Song et al. 2023). Soil cultivation media characteristics, such as physical structure, water retention, and nutrient availability, also critically influence cannabis plant performance (Burgel et al. 2020; Schober et al. 2023). SDI modifies these soil conditions by maintaining a more consistent moisture profile, minimizing evaporation, and promoting more efficient nutrient delivery within the root zone, which in turn can enhance root growth, nutrient uptake, and plant productivity, although the extent of the beneficial effect of SDI on these parameters is, to a certain degree, dependent on the soil’s physical properties like bulk density and clay content (Camp et al. 2000; Patel and Rajput 2008; Fan and Li 2018). Additionally, the absence of topsoil wetting, especially in foil tunnel cultivation systems, can lead to the increased accumulation of salts near the surface, which can result in adverse growing conditions for cannabis (Camp et al. 2000; Beheshti et al. 2023).

Therefore, understanding the specific interactions between SDI and soil-plant processes is crucial for optimizing cannabis cultivation outcomes under protected outdoor conditions. Furthermore, cannabis is often transplanted as clones with shallow rooting depth, which can limit the plant’s ability to access the water provided by the SDI during the crops’ establishment in the first two weeks after transplanting. Additionally, cannabis cultivation confronts significant challenges when plants are overwatered, leading to anaerobic conditions in the root zone, which can result in root rot and lower yields.

Thus, it remains unclear if SDI is an appropriate method for irrigating cannabis. This study compared the effects of nanofertilizers, SDI, and DI on water use efficiency, weed infestation, inflorescence yield, CBD concentration, and total CBD yield among three CBD-rich cannabis chemotypes III genotypes in a semi-controlled outdoor foil tunnel.

## Materials and methods

### Study site and foil tunnel setup

**Fig. 1:**
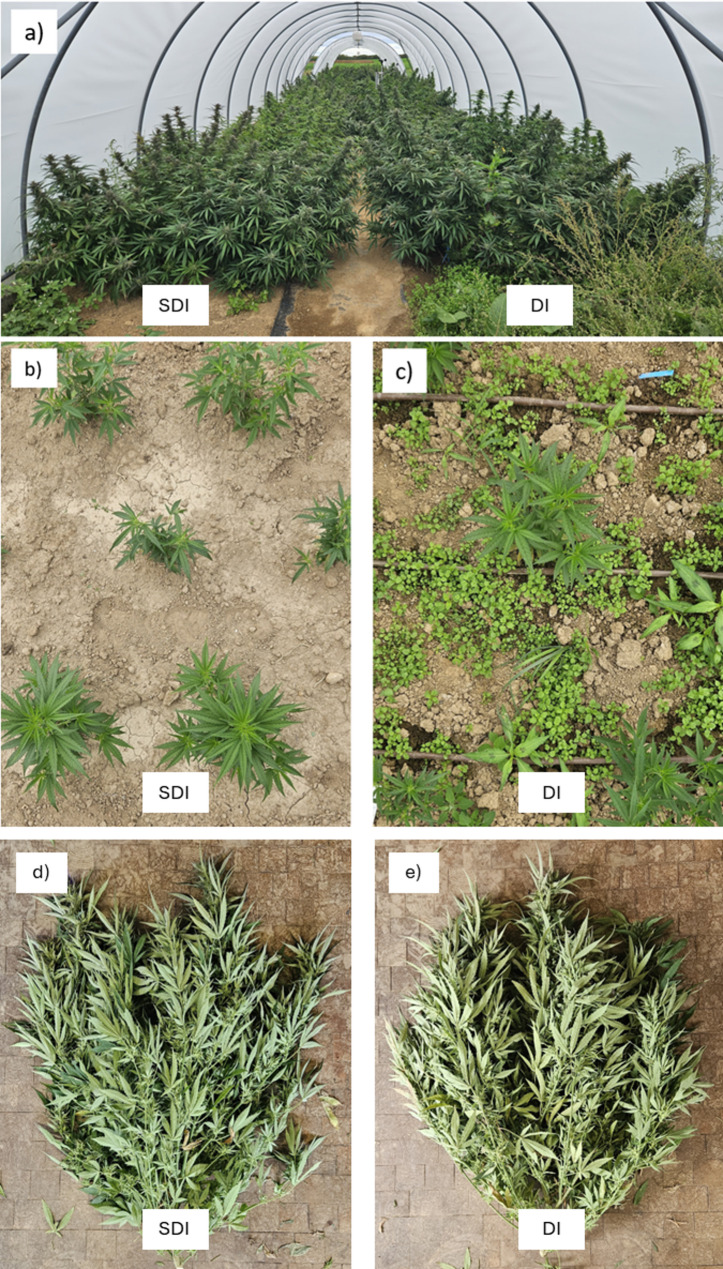
The experimental site at Ihinger Hof, Germany. (**a**) foil tunnel prior to final harvest (DAP 87). (**b**) plot irrigated with subsurface drip irrigation (SDI, DAP 24). (**c**) plot irrigated with surface drip irrigation (DI, DAP 24), (**d**) plant of the genotype Terra Italia grown with SDI at final harvest (DAP 98), (**e**) plant of the genotype Terra Italia grown with DI at final harvest (DAP 98)

**Fig. 2:**
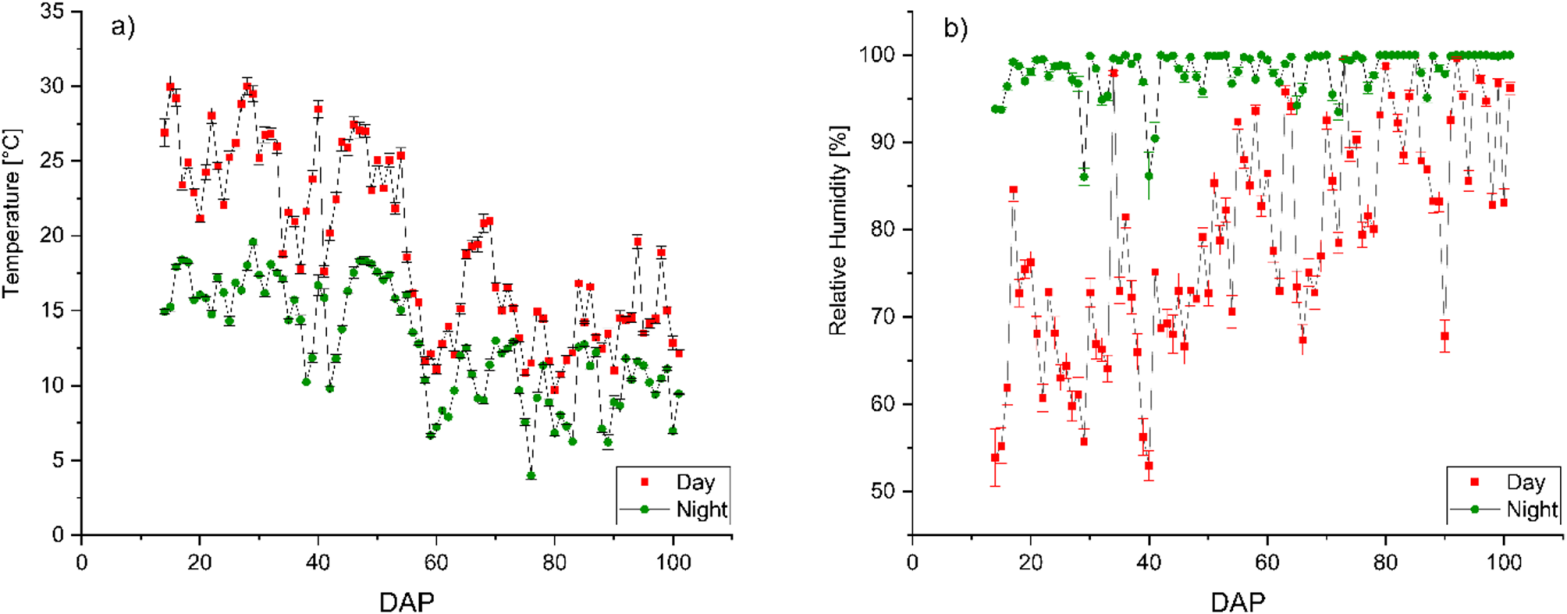
Mean day- and nighttime temperature [°C] (**a**) and mean day- and nighttime relative humidity [%] (**b**) in the foil tunnel over the growing period of cannabis plants from July-October 2024. Error bars represent the standard error of the mean. DAP = Days after planting

The experiment was conducted at the research station Ihinger Hof, University of Hohenheim, located near Renningen, Germany (48°44’33.0"N, 8°55’00.4"E) at an altitude of 478 m above sea level. The site is characterized by a mean annual temperature of 9.1 °C and an average annual precipitation of 697 mm. The average global irradiation of the growing period was 122.66 W m^− 2^, equivalent to 913.19 MJ m^− 2^ or 253.66 kWh m^− 2^, measured using an S-LIB-MOO3 Solar Radiation Smart Sensor (LI-COR Environmental, Lincoln, USA). Photosynthetically active radiation was 212.03 ± 2.98 µmol m^− 2^ s^− 1^, measured using an S-LIA-MOO3 PAR Smart Sensor (LI-COR Environmental, Lincoln, USA). Day and nighttime temperatures, as well as day and nighttime relative humidity over the growing period, are displayed in Fig. 2. Temperature and relative humidity inside the foil tunnel were measured using a Tinytag Plus 2– TGP-4500 (Gemini Data Loggers, Chichester, England). The soil at the site is classified as silty clay Luvisol with an organic matter content of 3.71% and a rooting depth of approximately 0.90 m. Prior to tunnel setup, the soil was plowed and tilled to ensure homogeneous soil conditions for each plot. A total of three foil tunnels measuring 15 m × 4.3 m were set up using a conventional polyethylene foil Lumisol Diffused AF (folitec Agrarfolienvertriebs GmbH, Westerburg, Germany) with a translucency of approx. 88% and a UVB transmission rate of 75–85%. Each tunnel was subdivided into two columns and two rows. Rows measured 11.5 m × 1.25 m. A walkway 1.5 m wide was prepared between the two rows in the center of each tunnel, using mulch foil, to facilitate access to the tunnel (Fig. 1a).

### Experimental setup

**Fig. 3:**
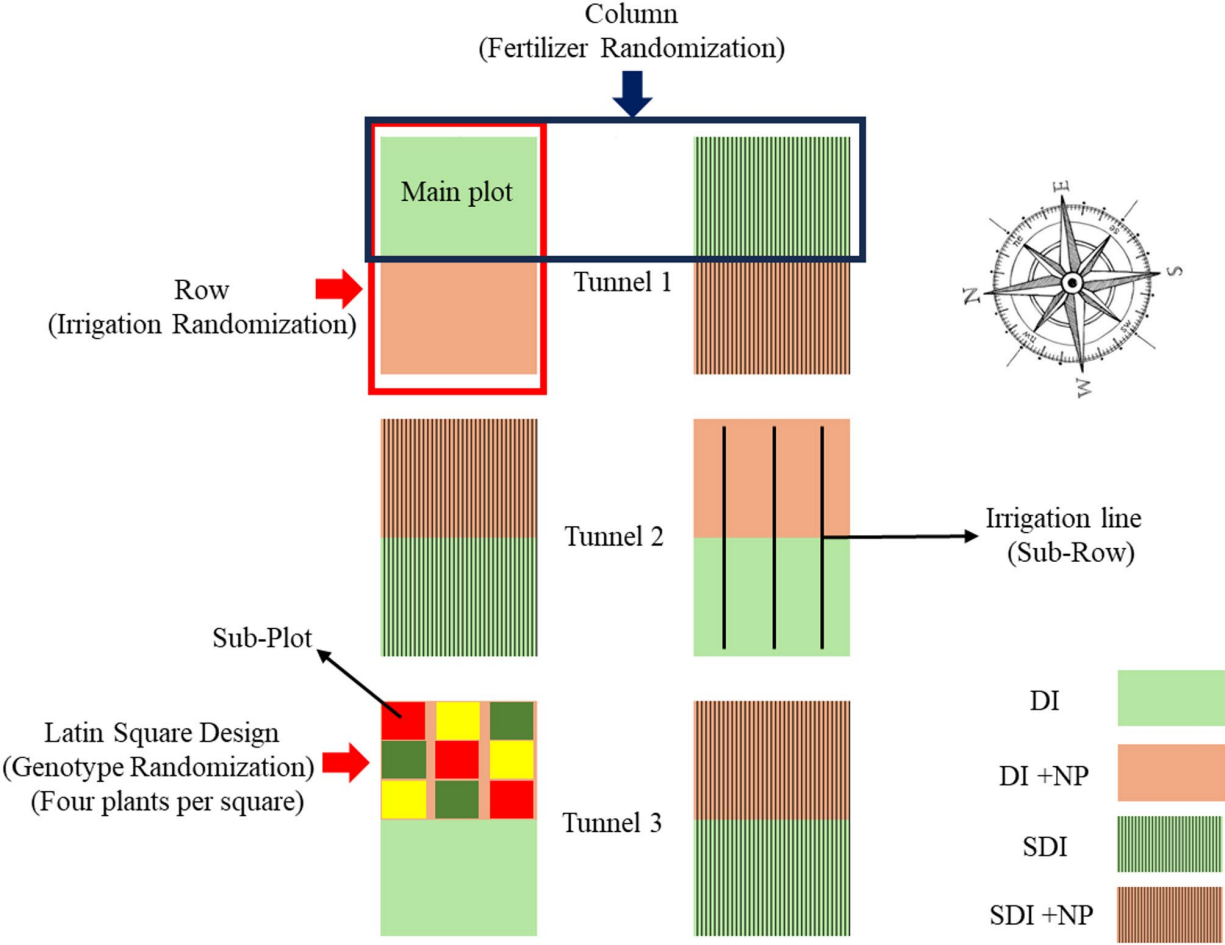
Schematic illustration of the experimental setup. DI = Surface Drip Irrigation, SDI = Subsurface Drip Irrigation, NP = Nanoparticles

Figure 3 illustrates the experimental setup graphically. The experiment was set up as a split-strip plot design, with each tunnel being one replicate. Each replicate was subdivided into four main plots arranged in two rows and two columns. Rows were used as randomization units for the irrigation method. Columns were used as randomization units for fertilizer treatment. Within each main plot, three genotypes were distributed to sub-plots of four plants according to a Latin square design. Before planting, all weeds were mechanically removed, and the sub-plots were tilled at a depth of 0.15 m. Soil samples from each sub-plot were collected and analyzed for mineral nitrogen content (N_min_). Afterwards, all sub-plots were individually fertilized with Calcium Ammonium Nitrate (CAN) (27% N (13.5% NO_3_-*N* + 13.5% NH_4_-N), 12% CaO) (EuroChem Agro GmbH, Mannheim, Germany) to a total amount of 270 kg N ha^− 1^. The experiment started on the 15th of July with the transplantation of the plants into the foil tunnel. Each row consisted of three sub-rows. Plants were planted in a triangular system to maximize space utilization, resulting in a planting density of five plants m^− 2^. This resulted in 72 plants per replicate and irrigation treatment. After transplanting, plants were manually watered twice at 17 L m^− 2^ to ensure rooting and plant establishment in the first two weeks.

### Treatments

#### Fertilizer treatment

To compare the effect of nanostructured fertilizer and biostimulants on plant growth and yield development, spherical, nonvalent nanoparticles of elemental silver (AgroArgentum), copper (AgroCyprum), and iron (AgroFerrum), provided by B + H Solutions GmbH (Remshalden, Germany), were applied weekly through the irrigation system. The application rates were 100 ml ha ^− 1^ for silver and 50 ml ha^− 1^ each for copper and iron, corresponding to the recommended dosages provided by the manufacturer. The proportional doser Dosatron D3RE2 (DOSATRONIC GmbH, Ravensburg, Germany) was utilized to inject the nanoparticle suspension into the system to ensure proper mixing of the nanoparticles into the fertigation suspension. Nanoparticle application was stopped 14 days prior to the final harvest.

#### Irrigation treatments

An ecoTube irrigation tube (ecoTube GmbH, Beselin, Germany) was installed at a depth of 0.35 m in the SDI treatment using a drainage plow to minimize soil disturbance. The distance between each row was 0.50 m. The ecotube system is built from recycled car tires and features a microporous structure that releases water at pressures exceeding 0.1 bar. The specified water release rate is 1.3 L per meter hour^− 1^. The DI system consisted of a Netafim Unitechline (Netafim, Tel Aviv, Israel), which offers a water release rate of 1.6 L per dripper per hour and a dripper spacing of 0.30 m. Both irrigation treatments were monitored using Gardena soil moisture sensors (Gardena, Ulm, Germany) installed at a depth of 0.25 m and linked to an irrigation computer (MultiControl Duo, Gardena, Ulm, Germany) to automate the irrigation process fully. Irrigation started once soil moisture dropped below 50% field capacity. The volume of water applied was recorded individually for every irrigation treatment via flow meters and measured regularly.

#### Irrigation amount

Because the tunnel was open on each short side, the length of the tunnel was not entirely utilized for the experiment due to possible changes in microclimate. Therefore, the length of the foil tunnel area was shortened by 1.75 m on each side. Irrigation tubes, however, were installed over the full length of the tunnel, inflating the actual irrigation amount per plot as parts of the tunnel were irrigated where no plants were growing. Therefore, the liter m^− 2^ values were adjusted to the actual irrigated plot size by the following equation:

$$\:{IA}_{hijklm}=\frac{{{IA}_{total}}_{hijklm}}{{{A}_{total}}_{hijklm}}\cdot\:{P}_{hijklm}$$

where $\:{IA}_{hijklm}$ is the volume of water in liters applied to the *hijklm*^th^ plot, $\:{{IA}_{total}}_{hijklm}$ is the total volume of water in liters applied at the experimental sites including areas with no plants, $\:{P}_{hijklm}$ is the actual size of the plot in m², and $\:{{A}_{{total}_{hijklm}}}_{}$ is the size of the irrigated area in m².

#### Irrigation water use efficiency

The Irrigation Water Use Efficiency (IWUE) was calculated as the slope of the relationship between the irrigation amount (covariate) and yield (response variable). The irrigation treatment was replaced with treatment-specific regression on the total amount of water irrigated.

For clarity, this can be expressed mathematically as:

$$\:IWUE=\frac{\varDelta\:Yield}{\varDelta\:Irrigation\:Amount}$$,

where ΔYield represents the change in yield, and ΔIrrigation Amount represents the change in the irrigation amount. Note that the irrigation amount was measured just once per irrigation treatment and replicate. Therefore, *p*-values for all tests, excluding irrigation treatments, were identical to those observed for yield, but the estimates and their interpretation changed.

### Genotypes and propagation

The experiment included three chemotype III genotypes: Kanada (AI Fame, Switzerland), Terra Italia (Female Seeds, Netherlands), and the auto-flowering genetic FED (AIFame, Switzerland). Of each genotype, 144 cuttings were cut from mother plants previously cultivated in the greenhouse of the University of Hohenheim. The base of each cutting was dipped in a 2% indole-3-butyric acid powder (Rhizopon AA2%, Rijndijk, Netherlands) to facilitate rooting and placed into 4 × 4 cm² eazyPlugs (HGA Garden B.V., Goirle, Netherlands) which were hydrated with regular tap water at a pH of 6.2. The cuttings were transferred into propagation tents equipped with ultrasonic humidifiers and ventilators to ensure proper air circulation. Relative humidity was maintained between 80% and 95%, with a temperature range of 22 °C to 27 °C. After two weeks, all cuttings were sufficiently rooted and transplanted into 0.5 L pots using a 10:1 (v/v) mixture of Substrate 5 (Klasmann-Deilmann GmbH, Geeste, Germany) and Perligran Extra (Knauf, Iphofen, Germany). Two weeks after transplanting, pots were thoroughly rooted and ready to be transplanted into the foil tunnel. Before planting, the apical meristem was removed above the fifth internode to break apical dominance and control the number of internodes. According to a Latin square design, genotypes were randomized to a grid of 3 × 3 plots within the main plots.

### Destructive measurements

Destructive measurements of all tested chemotypes were conducted six times during the growing season, at 24, 38, 59, 73, 87, and 101 days after planting (DAP), consisting of three replicates per genotype and treatment. On each sampling date, plant height was recorded, and the plants were cut off at the soil level. The above-ground biomass was separated into leaves, stems, and inflorescences to determine the biomass of each fraction. This was done for all genotypes individually. After measuring the fresh weight, each biomass fraction was bagged separately and dried in drying chambers at 30 °C for 14 days to estimate dry weight. Inflorescences were ground into a homogenous powder using an ultra-centrifugal mill (Type ZM 200; Retsch, Haan, Germany). The residual moisture of every inflorescence sample was determined using a moisture analyzer (DBS 60 − 3; Kern and Sohn GmbH, Balingen, Germany). Samples were stored in darkness until HPLC analysis.

#### Weed biomass

The weed biomass was recorded in all irrigation treatments at 24 DAP. Weeds were removed manually from each main plot. The fresh weight was recorded and then dried at 100 °C for 24 h to estimate the dry biomass.

#### Cannabinoid analysis

The inflorescence samples underwent analysis using high-performance liquid chromatography (HPLC), the established standard for cannabinoid quantification. This analysis followed the protocols outlined by Crispim Massuela et al. (2022). For cannabinoid extraction, approximately 100 ± 10 mg of ground, dried inflorescences were combined with 100 mL of a solvent composed of 90% methanol and 10% chloroform (v/v) and treated in an ultrasonic bath for 30 min at 40 °C. After cooling, the solution was filtered through 0.45 μm Polytetrafluorethylene (PTFE) syringe filters (Macherey-Nagel GmbH & Co. KG, Düren, Germany) into HPLC vials before being injected into the HPLC system (1290 Infinity II LC System, Agilent, Santa Clara, CA, USA). This system is equipped with an autosampler, a quaternary pump, and a diode-array spectrophotometer (DAD), set to operate at a detection wavelength of 230 nm.

Chromatographic separation was achieved using a Nucleosil 120-3 C8 column (125 mm × 4 mm i.d., 3.0 μm) paired with an EC 4/3 Nucleosil 120-3 C8 guard column (Macherey-Nagel, Oensingen, Switzerland). The mobile phase comprised HPLC-grade methanol (Solvent A) and 0.1% acetic acid in HPLC-grade distilled water (Solvent B; Sigma-Aldrich, Saint Louis, MO, USA), flowing at a constant rate of 0.7 mL min^− 1^ with a gradient elution mode. The injection volume was set at 10 µL, with a total run time of 27 min.

Integration of targeted peaks utilized analytical reference standards for CBD (C-045) and CBDA (C-144) (Sigma-Aldrich, Darmstadt, Germany), while data analysis was conducted with ChemStation for LC Rev. B.04.03-SP2 software (Agilent, Santa Clara, CA, USA). Calibration curves were created from diluted standard solutions, demonstrating a coefficient of determination of 1.0 for both CBD and CBDA. The limit of detection for these compounds was established at 0.0015%.

#### CBD concentration

The total CBD concentration of each genotype × irrigation × fertilizer treatment was calculated using the following equation:

$$\begin{array}{l}\:{CB{D}_{total}\left(\%\right)}_{hijklm}=\\{CBD}_{hijklm}+{CBDA}_{hijklm}\cdot\:0.877\end{array}$$

Where

$\:{CB{D}_{total}\left(\%\right)}_{hijklm}$, $\:{CBD}_{hijklm}$, and $\:{CBDA}_{hijklm}$ are the corresponding values of the $\:h{ijklm}^{th}$ plot. The factor 0.877 was applied to consider the differing molar masses of the acid and neutral form.

### Statistical model and analysis

Data from the final harvest were analyzed using the following mixed model

$$\begin{array}{l}\:{y}_{hijklm}=\mu\:+{t}_{h}+{c}_{hi}+{r}_{hj}+\\{mp}_{hij}+{sc}_{hijl}+{sr}_{hijm}+{\tau\:}_{i}+\\{\phi\:}_{j}+{\rho\:}_{k}+{\left(\tau\:\phi\:\right)}_{ij}+{\left(\tau\:\rho\:\right)}_{ik}+\\{\left(\phi\:\rho\:\right)}_{jk}+{\left(\tau\:\phi\:\rho\:\right)}_{ijk}+{e}_{hijklm}\end{array}$$,

where $\:{y}_{hijklm}$ is the observation of irrigation treatment *i*, fertilizer treatment *j* and genotype *k* in column *i* and row *j* of tunnel *h* measured in sub-column *l* and sub-row *m*, $\:\mu\:$ is the intercept, $\:{t}_{h}$, $\:{c}_{hi}$, $\:{r}_{hj}$, $\:{sc}_{hijl}$, $\:{sr}_{hijm}$, and $\:{mp}_{hij}$ are the block effects of tunnel *h* (assumed as fixed), column *i* and row *j* in tunnel *h* and sub-column *l* and sub-row *m* within a row-column-combination as well as the row-column-combination denoted as main-plot (all assumed as random), $\:{\tau\:}_{i}$, $\:{\phi\:}_{j}$, and $\:{\rho\:}_{k}$ are the fixed effects of irrigation treatment *i*, fertilizer treatment *j* and genotype *k*, $\:{\left(\tau\:\phi\:\right)}_{ij}$, $\:{\left(\tau\:\rho\:\right)}_{ik}$, $\:{\left(\phi\:\rho\:\right)}_{jk}$, and $\:{\left(\tau\:\phi\:\rho\:\right)}_{ijk}$ are random interaction effects of the corresponding main effects and $\:{e}_{hijklm}$ is the confounded effect of plot and error associated with $\:{y}_{hijklm}$. The terms $\:{c}_{hi}$ and $\:{r}_{hj}$ serves as randomization units for irrigation and fertilizer treatment, $\:{mp}_{hijlm}$ serves as main-plot of the split-plot design. For IWUE, $\:{\tau\:}_{i}$ was replaced by $\:{\beta\:}_{i}{x}_{hijklm}$, where $\:{\beta\:}_{i}$ is the irrigation treatment specific slope of yield on the amount of irrigated water $\:{x}_{hijklm}$. For weed biomass, data were only measured per main-plot. Therefore, the model can be simplified by dropping all effects including the index *k*, *l* and *m* from the model. In this case, $\:{mp}_{hij}$ serves as residual error. For traits that were measured repeatedly during the experiment, time was added as fixed effect to the model. Additionally, all fixed effects were added as interaction effects with time. Random effects were crossed with time and a first order auto-regressive variance covariance structure was assumed. Due to convergence problems, the variance-covariance structure was afterwards simplified to compound symmetry for all effects except the error. For all analyzed traits, residuals were checked graphically via residual plots. In case of deviations, data were logarithmically transformed prior to analysis. In this case, means were back-transformed for presentation purpose. Standard errors were back-transformed using the delta method. Treatment effects were tested for significance and multiple mean comparisons were performed for significant effects only using Fisher´s LSD test. Results were finally presented via letter display. Additionally, special contrasts of interest were tested using estimate statements. E.g. for flower depending traits, the time-by-genotype classification was unbalanced due to different vegetation length. In this case, marginal means for irrigation were not estimable but contrasts of irrigation treatment levels for a core set of genotype-by-time combinations can be estimated.

For transparency purpose, simple means were additionally calculated independent on significance of F tests. These means were compared pair-wise. To account for multiple testing, t-tests were adjusted by the method of Bonferroni.

#### Graphing

Graphs were created using the graphing software OriginPro 2024 (OriginLab Corp., Northampton, Massachusetts, USA).

## Results and discussion

The nano-fertilizers showed no statistically significant impact. Furthermore, for yield and most traits, no significant interactions were found (Table 1). In these cases, marginal means should be estimated across levels of fertilizer (Heiman 2001; Toutenburg, 2002; Welham et al. 2015). Marginal means for inflorescence yield and CBD concentration are displayed in Fig. 6. However, for additional information and for reasons of transparency, means for non-significant interaction term nanofertilizer × irrigation treatments were calculated for inflorescence yield and CBD concentration, too. They were presented analogous to Fig. 6 (Fig. 7). Figure 7 highlights the insignificant effect of the fertilizer treatment on the tested parameters. Note that means in Fig. 6 were based on six observations. In contrast, means in Fig. 7 are based on three obsevatiuons causing a loss of power to detect significant differences.

### Water consumption

**Fig. 4:**
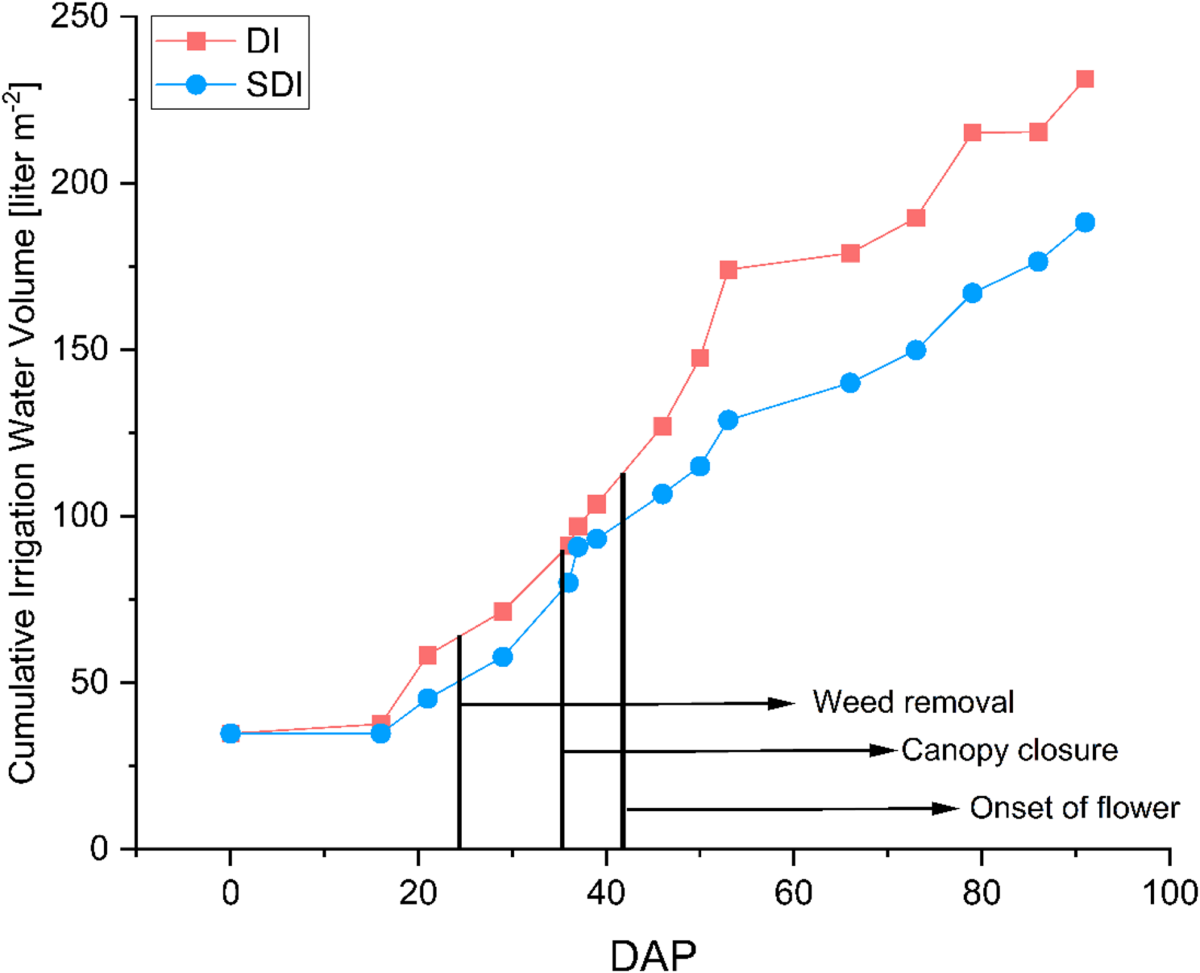
Cumulative irrigation water volume (liter m^-2^) for drip irrigation (DI nI) and subsurface drip irrigation (SDI l) treatments over the growing period. DAP = Days after Planting

Figure 4 illustrates the cumulative volume of irrigated water for each irrigation method during the growing period. Compared to DI, SDI resulted in an 18.6% reduction in water consumption from 231.23 L m^− 2^ for DI and 188.19 L m^− 2^ for SDI, resulting in a total irrigation amount of 9971.6 L and 8116.08 L for DI and SDI, respectively. Figure 4 presents the irrigation water amount m^− 2^ as the cumulative water use over the growing period. The amount of irrigated water is consistently lower throughout the entire growing period in SDI compared to DI. The highest significant difference in applied water occurred from DAP 36 to DAP 52, where the irrigation water applied was reduced by 51% in SDI compared to DI. In Fig. 2, it becomes visible that during this time, temperatures increased.

These findings align with the established knowledge regarding the water-saving potential of SDI compared to DI. For instance, Valentín et al. (2020) examined the WUE of conventional sprinklers, DI, and SDI in a *Zea mays* production system in southeastern Spain, where the use of SDI resulted in a 30% reduction in water consumption compared to DI. Particularly during the initial plant development phase, when soil evaporation constitutes a significant portion of total evapotranspiration due to relatively low canopy coverage, evaporation losses decreased by over 60% in SDI. Similar results were reported by Hassanli et al. (2009), who found a reduction in water usage between 6% and 16% for SDI compared to DI in an open-field maize cultivation system. Najafi and Tabatabaei (2007) observed reductions in irrigation water amounts of 9% and 46% for tomato and eggplant production, respectively, when using SDI compared to DI and flood irrigation. Furthermore, Martínez and Reca (2014) indicated in their study that by using SDI, the volumetric soil water content is higher and remains more stable when compared to DI as a result of reduced capillary flow to the soil surface and reduced evaporation losses, which led to a higher soil water content in the root zone, especially when combined with deficit irrigation. Similar to our study, irrigation intensity and frequency were controlled via soil moisture sensors; it can be assumed that soil water content was also maintained in a suitable range.

### Weed biomass

**Fig. 5:**
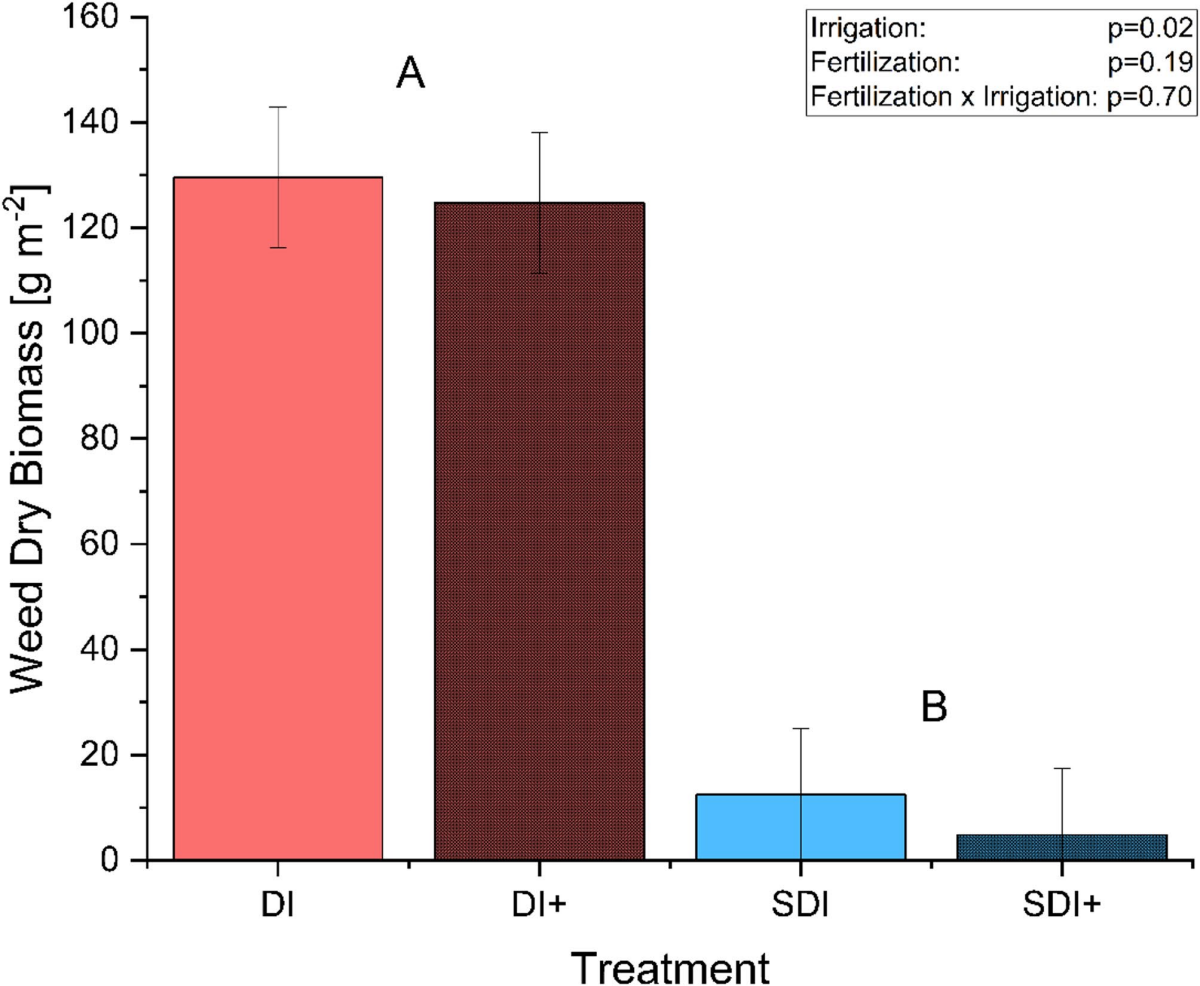
Weed biomass in g DM m^− 2^ for plots irrigated with surface drip irrigation with (DI+) or without (DI) nanoparticle application and plots irrigated with subsurface drip irrigation with (SDI+) or without (SDI) nanoparticle application 24 days after planting (DAP). Error bars represent the standard error. As Irrigation-by-fertilizer interactions were non-significant, letters indicate results of irrigation main effect comparison, where different letters represent differences between irrigation main effects according to F and t test at a = 0.05. Note that results from multiple irrigation-by-fertilizer mean comparisons were non-significant due to loss of power

Figure 5 shows the weed biomass dry matter m^−^² at DAP 24. Furthermore, pictures of the respective plots are presented in Fig. 1b and Fig. 1c. Again, no statistically significant effects of nanofertilization on weed density were found (*p* = 0.19). Weed biomass showed a reduction of 93.21%, significantly lower in SDI compared to DI (*p* = 0.0224). This beneficial effect of SDI has also been shown for other crops in other studies. Sutton et al. (2006) compared weed density in tomato production using either SDI or furrow irrigation, combined with or without herbicides, in standard or conservation tillage. Weed densities were reduced by over 98% in SDI, regardless of tillage method or herbicide usage. Most impressively, they also discovered that weed densities were lower in the SDI conservation tillage without herbicide treatment than in the furrow-irrigated standard tillage with herbicide application. Kakraliya et al. (2024) observed a similar effect in a rice-wheat cultivation system, where the adoption of SDI reduced weed density by up to 72.9%, depending on the tillage combination. It is hypothesized that the reduction in weed biomass under SDI is mainly due to the drier topsoil conditions and, therefore, limited water availability, as weeds like *Stellaria media* or *Solanum nigrum* do not root as deep as cannabis and thus compete for different water sources (Van Delden et al. 2002).

The reduction in weed biomass reduces competition for resources between weed and crop plants and can also decrease the man-hours per hectare needed for weed management while increasing economic profitability (Sutton et al. 2006). There is a prevalent belief that cannabis possesses a competitive advantage over common weed species. This might be true for some genotypes but could be inaccurate for shorter, less vigorous varieties. As a result, there are few, if any, herbicides approved for use in cannabis cultivation systems depending on the producing country, greatly restricting the available options for weed management (Sandler and Gibson 2019; Gitsopoulos et al. 2024). Furthermore, resource input in cannabis cultivation is already substantial. A reduction in weed density by default as a side-effect of the change in the irrigation system not only increases water use efficiency but also eliminates a possible source of product contamination, which is especially important for crops used for medicinal purposes (Zheng et al. 2021; Buirs and Punja 2024; Desaulniers Brousseau et al. 2024). Additionally, SDI is often combined with conservation tillage that, compared to conventional tillage, has many ecological benefits such as improved soil organic matter, higher carbon sequestration, less soil erosion, decreased soil compaction, increased soil microbial activity, as well as reduced workload, and reduced greenhouse gas emissions SDI, combines the beneficial effects of both systems increasing the resource use efficiency of medicinal cannabis production (Sutton et al. 2006; Lehman et al. 2015; Rahman et al. 2021; Ogieriakhi and Woodward 2022).

### Yield parameters

#### Irrigation technique

**Fig. 6:**
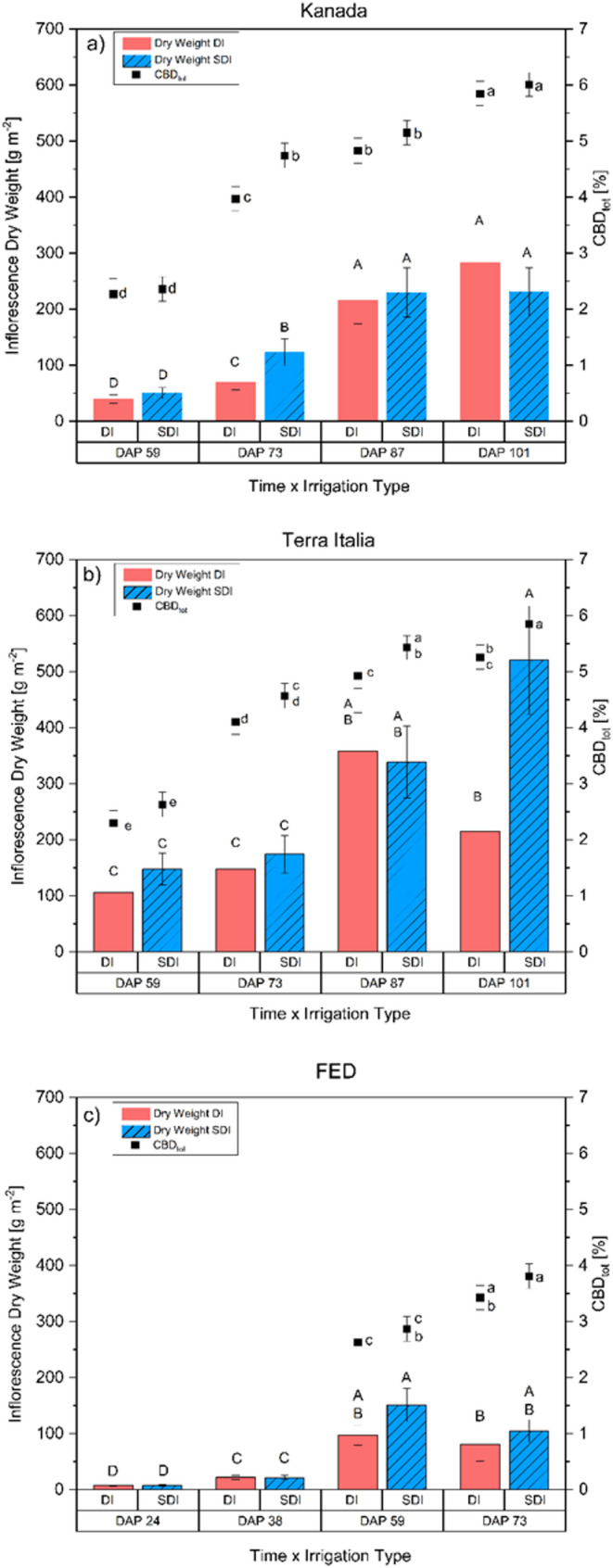
Inflorescence dry weight [bars] (g m^− 2^) and CBD concentration [squares] (CBD_tot_) of three chemotype III cannabis (Cannabis sativa L.) genotypes Kanada (a), Terra Italia (b) and FED (c) subjected to either surface drip irrigation (DI) or subsurface drip irrigation (SDI) depending on different sampling dates (DAP). Capital letters indicate differences in inflorescence yield within a genotype. Lowercase letters indicate differences in CBD-concentration within a genotype. Means with at least one identical lowercase letter are non-significant different from each other (a = 0.05, Fisher´s LSD test)

For the general comparison of the effect of irrigation treatment on inflorescence yield, only data for the genotypes Kanada and Terra Italia for DAP 59, 73, 87, and 101 were used. A comparison of all genotypes and sampling dates was not possible due to the different dates and a significantly shorter growing period of FED. Yield data indicated that plants irrigated with SDI had a 5% significantly higher inflorescence yield than plants irrigated with DI (*p* = 0.027). Note that there is also a significant interaction of Genotype × Irrigation × Sampling time (*p* = 0.028). These interactions are based on varying irrigation treatment effects across genotype-by-sampling point combinations. However, in 10 out of the 12 comparisons, yield under SDI was non-significant or significantly higher than DI, while it was non-significantly lower at two-time points (Fig. 6a-c). For FED, there was a visible but not statistically significant decline in inflorescence yield from DAP 59 to DAP 73, regardless of irrigation regime. During sampling, it was noticeable that this genotype was nearly fully mature on DAP 59. Note that this auto- flowering genotype matures much faster than its photoperiodic-dependent counterparts and is typically fully mature between 60 and 65 DAP. The decline in yield can be explained by the number of flowers infected with *Botrytis cinerea*, which increased from DAP 59 to DAP 73 and caused the decrease in inflorescence yield (Punja and Ni 2021; Buirs and Punja 2024)Fig. 6c). A similar effect was found for the genotype Kanada (Fig. 6a) and Terra Italia (Fig. 6b), where inflorescence dry weight was higher in four out of five sampling dates for each genotype. However, this lacked statistical significance for most sampling dates. Interestingly, at the last sampling date, the inflorescence yield of Kanada was non-significantly lower in SDI compared to DI, whereas it was significantly higher in Terra Italia. Differing reactions of different cannabis genotypes to growing conditions and environmental factors are well known (Campbell et al. 2019; Babaei and Ajdanian 2020; Haworth et al. 2024). Additionally, in past experiments investigating the impact of water deficit on these two genotypes, it was found that both genotypes react significantly different to watering regimes, providing a possible explanation for the contrasting reactions (unpublished data). However, given that this differing reaction was only observed at one sampling date for each genotype and statistical significance was not found for both cases, a random interaction seems most plausible.

When considering the CBD concentration of the plants, a similar tendency for non-significantly higher CBD concentrations under SDI compared to DI became visible. When comparing all genotypes and sampling dates, plants irrigated with SDI had a 9% higher CBD concentration than those irrigated with DI, with the difference being statistically significant (*p* < 0.001). This is particularly interesting as CBD concentrations usually decline with increasing inflorescence yield as part of a dilution effect (Shiponi and Bernstein 2021b; Massuela et al. 2023). One possible explanation is that the reduced competition for resources, stemming from decreased weed growth and more stable soil water content associated with using SDI, created a more suitable environment. This environment can lead to increased yields and higher cannabinoid concentrations to an extent where the dilution effect was compensated (Martínez and Reca 2014; Bernstein et al. 2019; Massuela et al. 2023).

#### Nanofertilizer

**Fig. 7:**
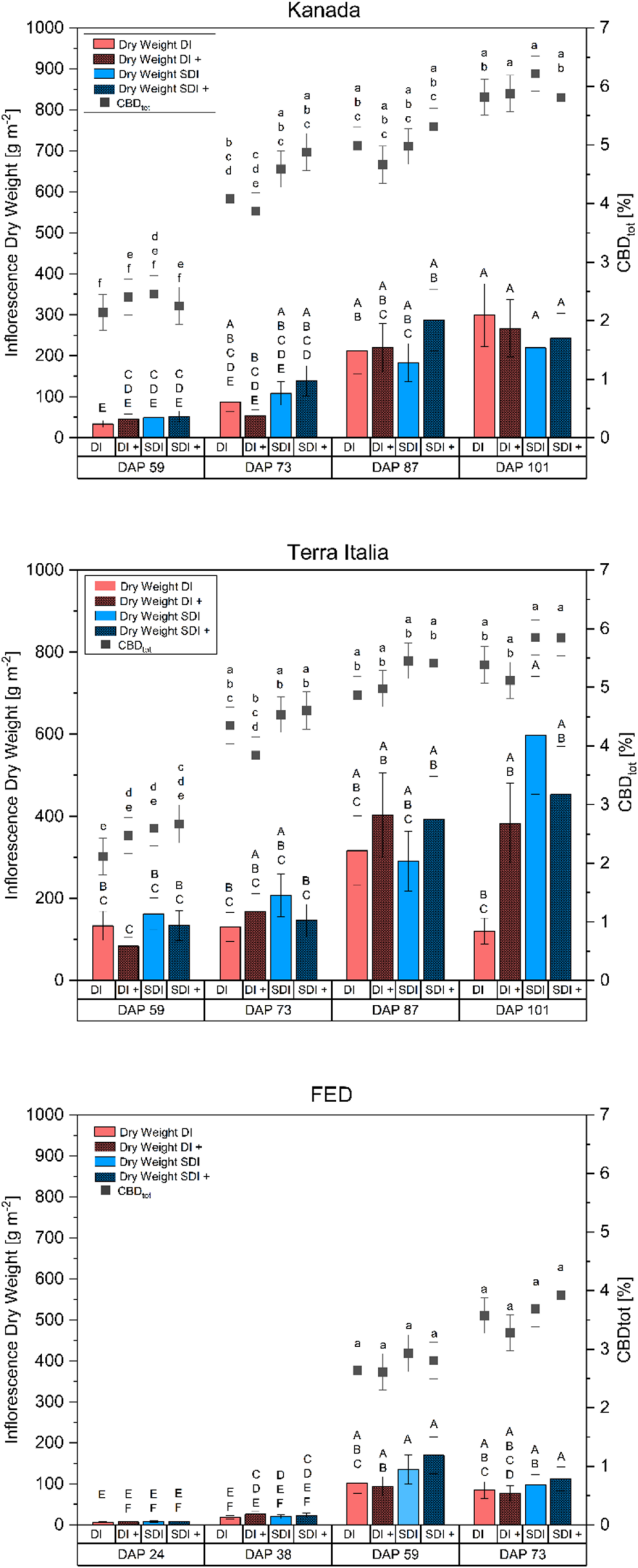
Inflorescence dry weight [bars] (g m^− 2^) and CBD concentration [squares] (CBD_tot_) of three chemotype III cannabis (*Cannabis sativa* L.) genotypes Kanada (a), Terra Italia (b) and FED (c) subjected to either surface drip irrigation without nanofertilizer (DI) and with fertilizer (DI+) or subsurface drip irrigation without nanofertilizer (SDI) and with nanofertilizer (SDI+) depending on different sampling dates (DAP). Error bars represent the standard error of the mean. CBD means within a genotype with at least one identical lower-case letter were non significant different at Bonferroni adjusted a = 0.05. Dry weight means within a genotype with at least one identical upper-case letter were non significant different at Bonferroni adjusted a = 0.05. *n* = 3

For descriptive purposes, simple means of the inflorescence yield and CBD concentration are displayed for all treatment combinations (Fig. 7). Final inflorescence dry yield m^− 2^ ranged from 219.7 ± 54.1 g to 299.6 ± 76.5 g, from 120.5 ± 31.5 g to 597.7 ± 143.9 g and from 76.0 ± 19.3 g to 112.4 ± 29.1 for the genotypes Kanada, Terra Italia and FED, respectively. CBD_tot_ ranged from 5.8 ± 0.3% to 6.2 ± 0.3%, 5.1 ± 0.3% to 5.9 ± 0.3%, and 3.3 ± 0.3% to 3.9 ± 0.3%, for Kanada, Terra Italia, and FED, respectively. No statistically significant effects were found from applying nanofertilizers on inflorescence dry mass (*p* = 0.363), CBD concentration (*p* = 0.808), or total CBD yield (*p* = 0.232) in the three *Cannabis sativa* L. genotypes studied. Additionally, no significant interactions with the other variables tested were observed for these parameters. In contrast, for IWUE, significant three-way interactions were noted between genotype, irrigation method, and fertilizer type for both inflorescence and CBD yield. For IWUE, CBD concentration showed only a main effect for the irrigation method, which reached significance. The corresponding *p*-values for all tested parameters are shown in Table 1. These results support the existing literature, which emphasizes significant genotypic variability within *Cannabis sativa* L., leading to different physiological and agronomic responses to nutrient and water management techniques (Babaei and Ajdanian 2020; Sheldon et al. 2021; Massuela et al. 2023). Furthermore, the understanding of nanofertilizer effectiveness remains inconsistent, with little agreement, particularly in the field or similar settings where environmental variability complicates treatment results (Rizwan et al. 2021). Unlike bulk-material counterparts, the behavior and bioactivity of nanoparticles depend on physicochemical properties such as particle size, morphology, surface coating, and concentration, which significantly affect their interactions with plants, uptake processes, and overall effectiveness (Singh 2016; Khan et al. 2019; Ullah et al. 2023). Importantly, the same nanoparticle formulations can have varying effects on different crop species, indicating a pronounced crop-specific response that limits the generalization of findings (Lee et al. 2012; Farghaly and Nafady 2015). There are limited studies available on the effect of nanoparticles on cannabis, with, to our knowledge, none currently existing on the effect of silver nanoparticles, especially under field conditions. For example, Cahill et al. (2024) tested the effect of copper oxide nanoparticles (CuO-NPs) on the THC and CBD concentrations of two cannabis cultivars. They found that the application of CuO-NPs increased THC and CBD levels compared to copper bulk material in one cultivar, while the other cultivar remained unaffected. However, the authors did not provide any data on inflorescence or cannabinoid yield. Thus, it remains uncertain whether the increase was due to an absolute increase in cannabinoid concentration or because of a concentration effect. Furthermore, they used foliar application in contrast to the soil application used in this study. It has been demonstrated in previous studies that the application method significantly impacts the efficacy of nanoparticle application (López-Luna et al. 2023), thereby limiting the transferability of the results presented by Cahill et al. (2024). Deng et al. (2022) studied the effect of Fe_3_O_4_ nanoparticles on cannabis clones in a hydroponic cultivation system and found that THC values decreased significantly compared to the untreated control. However, both studies mentioned above used oxidized, single-element nanoformulations, which contrasts with the combined application of elemental silver, copper, and iron NPs used in this study. They either employed a different application method or cultivation system. As stated above, those factors greatly impact the effect of the tested particles. Thus, studies that do not use comparable nanoparticle formulations, crop species, application methods, or cultivation systems possess limited predictive value and should be interpreted cautiously. Given the current lack of field data and uncertainties regarding the long-term environmental behavior of nanoparticles, more research is essential to thoroughly assess their agronomic suitability, environmental safety, and functional reliability, especially under field conditions.

**Table 1:** Statistical significance (*p*-values) of fixed main effects and their interactions from the fitted linear mixed-effects model. The table summarizes *p*-values for F-tests for the traits inflorescence yield, CBD concentration (CBD_tot_), CBD yield (CBD_yield_), and their respective irrigation water use efficiencies (IWUE). Cells marked “–” indicate that the test was not performed for that corresponding trait. Values in bold denoted F-tests for which mean comparisions should be done

Effect	Inflorescence yield	CBD_tot_	CBD_yield_	IWUE Inflorescence Yield	IWUE CBD_tot_	IWUE CBD_yield_
Genotype	< 0.001	0.16	< 0.001	0.09	0.003	0.12
Irrigation	0.027	**< 0.001**	0.011	0.13	**< 0.001**	0.11
Time	< 0.001	< 0.001	< 0.001	-	-	-
Irrigation × Time	0.64	0.54	0.73	-	-	-
Irrigation × Genotype	0.58	0.52	0.48	0.005	0.09	0.002
Genotype × Time	0.0002	**< 0.001**	< 0.001	-	-	-
Genotype × Irrigation × Time	**0.028**	0.56	**0.007**	-	-	-
Fertilization	0.26	0.81	0.23	0.21	0.57	0.23
Fertilization × Genotype	0.82	0.93	0.89	0.15	0.30	0.12
Fertilization × Time	0.72	0.96	0.33	-	-	-
Fertilization × Genotype × Time	0.37	0.83	0.31	-	-	-
Fertilization × Irrigation	0.57	0.47	0.88	0.08	0.96	0.06
Fertilization × Irrigation ×Time	0.46	0.27	0.26	-	-	-
Fertilization × Irrigation × Genotype	0.09	0.93	0.08	**0.022**	0.62	**0.022**
Fertilization × Irrigation × Genotype × Time	0.15	0.52	0.14	-	-	-

### Water use efficiency

**Table 2:** Irrigation water use efficiencies (IWUE) for the inflorescence yield, CBD concentration (CBD_tot_), and CBD yield m^-2^ of two chemotype III genotypes, Kanada and Terra italia. (DI = Drip irrigation, DI + = Drip irrigation with nps, sdi = subsurface drip irrigation, SDI + = subsurface drip irrigation with NPs). Means with at least one identical letter are not significantly different according to Fisher´s LSD test at α = 0.05

Irrigation Type	Genotype	IWUE Inflorescence yield	IWUE CBD_tot_	IWUE CBD_yield_
DI	Kanada	1.26 ± 0.31 b	0.026 ± 0.001 b	0.07 ± 0.02 b
	Terra Italia	0.52 ± 0.13 c	0.022 ± 0.001 cd	0.03 ± 0.01 c
DI +	Kanada	1.10 ± 0.28 b	0.025 ± 0.001 cb	0.06 ± 0.02 b
	Terra Italia	1.62 ± 0.40 ab	0.022 ± 0.001 d	0.08 ± 0.02 b
SDI	Kanada	1.19 ± 0.30 b	0.033 ± 0.002 a	0.07 ± 0.02 b
	Terra Italia	3.17 ± 0.80 a	0.031 ± 0.002 a	0.19 ± 0.04 a
SDI +	Kanada	1.28 ± 0.32 b	0.031 ± 0.001 a	0.07 ± 0.02 b
	Terra Italia	2.46 ± 0.62 ab	0.031 ± 0.001 a	0.14 ± 0.03 a

Table 2 displays the IWUE of inflorescence yield, CBD concentration, and CBD_yield_ m^− 2^. There was a highly significant interaction of Irrigation Treatment × Genotype × Fertilizer for inflorescence and CBD yield. The inflorescence yield IWUE was significantly higher in SDI than DI for Terra Italia but lacked statistical significance for Kanada, regardless of fertilization type. When irrigated with DI, the genotype Terra Italia expressed significantly higher IWUE in the nanoparticle fertilized treatment DI + than without nanoparticle application but remained lower than in SDI at both fertilizer treatments. IWUE of both genotypes did not differ significantly for DI. Similarly, as plants irrigated with SDI showed significantly higher CBD_tot_ values, the IWUE of CBD_tot_ was significantly higher in SDI than in DI, independent of the genotype and fertilization type tested. Here no interaction was found. In DI, the IWUE for CBD_tot_ of Kanada was significantly higher than that of Terra Italia, mainly because CBD_tot_ was higher in Kanada. However, this difference disappeared under SDI. Again, the large genotypic variance in cannabis, especially in relation to watering strategies and intensities, can provide a possible explanation for this difference and genotypic interaction (Duong et al. 2023). When looking at the IWUE of the CBD_yield_, the difference between the genotypes becomes most apparent. While the IWUE of Kanada did not differ between irrigation and fertilization treatments, Terra Italia had a significantly higher IWUE of CBD_yield_ in SDI than DI due to significantly increased CBD concentration and inflorescence yield.

These observations align with the current knowledge of yield development and water use efficiency under SDI for many plant species. For example, Hansona et al. (1997) observed similar yield levels for lettuce when irrigated with SDI compared to traditional furrow irrigation while decreasing the irrigation water amount by 25–32%, depending on the crop cycle. Furthermore, Bozkurt Çolak (2021) showed that the fruit yield of bell peppers irrigated with SDI was comparable to that irrigated with DI, but most interestingly, they also showed that the dry matter yield of plants irrigated with SDI under deficit irrigation had significantly higher yield compared to DI combined with deficit irrigation, implying a higher water use efficiency under water limiting conditions. Similar results were found for maize plants, where an SDI irrigation system led to an average reduction of 25% in irrigation water applied without compromising yield levels, drastically increasing water use efficiency (Valentín et al. 2020). Mattar et al. (2021) demonstrated that fresh tuber yield in potato plants irrigated with SDI was significantly higher than that of plants irrigated with DI. They hypothesized that this increase was due to a higher volumetric soil water content in SDI than in DI. This hypothesis was further supported by the observation that there was no significant difference in dry tuber weight between the two irrigation methods. This indicated that the tubers in the SDI treatment had an elevated water content, likely due to higher water availability. Furthermore, in a global meta-analysis of the published literature on SDI, Wang et al. (2022) found that SDI significantly increased the yield parameters of crops, fruits, and vegetables compared to surface watering methods. The positive effect was most pronounced when dripper spacing was < 0.25 m and used in soils with a medium texture and higher clay contents. As the SDI system used in this study was a perforated tube that emitted water over the entire tube length, a uniform water distribution can be assumed, meeting these criteria. Similarly, Guo et al. (2023) found in their meta-study on subsurface irrigation methods that yield increased by 5.96% and IWUE by 27.72% in SDI compared to DI. They also found that SDI proved to be most effective compared to surface watering methods when the soil bulk density was relatively high and had a finer texture, soil characteristics similar to those found at the study site in this research, which supports our results.

## Conclusion

This study demonstrated SDI’s beneficial effects on irrigation water amount, weed infestation, yield variables, and irrigation water use efficiency in an outdoor foil tunnel production system. Using SDI instead of DI reduced the amount of irrigation water by 18.6%, while weed infestation was 93.21% lower. The decreased competition for resources, resulting from reduced weed infestations, led to a 5% increase in inflorescence yield and a 9% higher CBD concentration in plants irrigated with SDI, significantly enhancing the obtainable CBD yield per square meter. The nanoparticle treatment did not significantly affect either inflorescence yield, CBD concentration, nor CBD yield. However, a three-way interaction of genotype × irrigation × fertilizer was found for the IWUE of inflorescence yield and CBD yield m^− 2^, indicating a genotypic variance in response to nanoparticle application in medicinal cannabis concerning water use efficiency. While outdoor production can help to reduce the high greenhouse gas emissions associated with indoor cannabis cultivation, it often does not adequately address the comparatively high-water usage. Implementing SDI in outdoor foil tunnel cultivation systems can lead to substantial water savings and significant reductions in weed infestations. Given that medicinal cannabis production is projected to increase substantially in the coming years, further research is needed to assess the suitability of more sustainable approaches for cannabis cultivation.

## Data Availability

The datasets analyzed during the current study are available upon reasonable request, and the custom code used in this study is available from the corresponding author upon reasonable request.

## Declarations

### Use of AI

The AI software Grammarly (Grammarly Inc., San Francisco, USA) was used to assist with grammar and language corrections. However, the authors solely generated all original content, ideas, and input text..
